# Dispersion of Cellulose Nanofibers in Methacrylate-Based Nanocomposites

**DOI:** 10.3390/polym15153226

**Published:** 2023-07-28

**Authors:** Valentina Cavallo, Sébastien Pruvost, Jean-François Gerard, Alberto Fina

**Affiliations:** 1 Université de Lyon, CNRS, Université Claude Bernard Lyon 1, INSA Lyon, Université Jean Monnet, UMR 5223, Ingénierie des Matériaux Polymères, CEDEX, F-69621 Villeurbanne, France; valentina.cavallo@insa-lyon.fr (V.C.); sebastien.pruvost@insa-lyon.fr (S.P.); 2 Dipartimento di Scienza Applicata e Tecnologia, Politecnico di Torino, V.le Teresa Michel, 5, 15121 Alessandria, Italy

**Keywords:** cellulose nanofibers, nanocomposites, methacrylic copolymer, solvent casting

## Abstract

Poly(methylmethacrylate-*co*-methacrylic acid) (PMMA-*co*-MAA) polymers were prepared via cobalt-mediated free radical copolymerization and were characterized after synthesis. The synthesis led to a 98.5% conversion and a final ratio between the two units, MMA/MAA, was equal to 63:37 mol%. PMMA-*co*-MAA was then used as a matrix for cellulose-based nanocomposites to tailor filler compatibility, thanks to the presence of carboxylic groups capable of generating strong H-bonds with the cellulose surface. Cellulose nanofibers (CNFs) were dispersed using a solution with a mixture of two solvents to tailor compatibility of both the components. For this purpose, CNFs were successfully re-dispersed in methanol using the solvent exchange method and tetrahydrofuran/methanol mixtures at different ratios were used for the preparation of the films. Fully transparent films of PMMA-*co*-MAA + CNF were prepared up to 15 wt% of CNF with a good dispersion in the matrix. This dispersion state leads to the reinforcement of the polymethacrylate matrix, increasing its tensile strength whilst preserving optical transparency.

## 1. Introduction

Cellulose is a bio-based polymer derived from abundant and renewable resources, which, in the last decade, has attracted considerable attention, thanks to its combination of unique properties including excellent mechanical and thermal properties and versatile surface chemistry [1,2,3]. As pristine cellulose is not processable in a molten state owing to its strong H-bound chains, its valorization was traditionally carried out via extensive chemical modifications to obtain thermoplastic polymers [2,4,5] or using fibers as reinforcing agents in polymers. The possibility to produce cellulose nanoparticles with a high aspect ratio, high crystallinity, low density and thermomechanical stiffness using this second approach attracted much attention [6,7]. Cellulose nanoparticles are a fascinating option for the preparation of “all-polymer” composites. This is particularly relevant for designing sustainable materials, including bio-based and biodegradable polymers. Therefore, nanocellulose has been widely proposed for designing nanocomposites for a large set of applications. For example, with the use of nanocellulose, reinforcement in transparent and lightweight materials, barrier agents for membranes, fully biodegradable nanocomposite films, nanocellulose-based scaffold for tissue regeneration were achieved [8,9,10,11].

Nanosized cellulose can be mainly divided into three categories: (i) cellulose nanocrystals (CNCs) characterized by a rod-like nanostructure with a high degree of crystallinity (50–90%), (ii) cellulose nanofibers (CNFs) with cross-section in the range of nanometers and lenght in there range of 1 to 10 μm, composed of both amorphous and crystalline phases, and (iii) bacterial nanocellulose (BNC) produced from bacteria as ribbon-shaped objects of 20–100 nm in length [11,12]. CNCs and CNFs are commonly produced from a chemical pre-treatment of cellulose (i.e., acidic hydrolysis, TEMPO-mediated oxidation, and enzymatic pre-treatment), aiming to release cellulose nanofibers from the other components initially present in the lignocellulosic biomass. The chemical treatments can be preceded and/or followed by a mechanical fibrillation (i.e., high-pressure homogenization, grinding and sonication) [13]. Unfortunately, processing of cellulosic fibers, and particularly nanocellulose, is extremely challenging, given the presence of strong H-bond interactions which cause poor compatibility with conventional thermoplastic polymers considered as a matrix. As a result preparation of cellulose nanocomposites via melt compounding [14,15,16] or solvent casting [6,8] is challenging.

In melt processing, the dispersion of nanofillers is obtained using an extruder and it can be either a batch or a continuous process. From an industrial point of view, the melt processing technique attracts much interest since it could be easily scaled up. The cellulose can be directly fed into the extruder, minimizing the risk of nanoparticles volatilization [6,14,16]. However, processing of dry nanocellulose in polymers is extremely challenging owing to the strength of interactions between cellulose nanoparticles, which are by far stronger than the interactions between cellulose and polymer. Indeed, agglomeration of cellulose nanoparticles related to drying processes, also known as hornification, easily takes place during manufacturing processes where water removal is involved. In these conditions, water removal modifies the microstructure of cellulosic fibers and results in its lower capacity to retain water and the requirement of a higher amount of energy for re-dispersion [17,18]. To weaken the interactions between cellulose, suspensions of cellulose in water can be directly added during the melt mixing. Upon heating, water is removed, while cellulose is mixed within the polymer, possibly establishing matrix–nanoparticle interactions to limit its self-aggregation [19]. Chemical functionalization of cellulose [20,21] or the use of compatibilizing agents [22] may be considered to enhance interactions with the polymer and to reduce self-interactions. Despite conducting extensive investigations on these routes, obtaining a well-dispersed nanocellulose in a polymer remains challenging in many matrices and typically requires the addition of a highly polar compatibilizing agent [15,20,23]. This is particularly relevant for polyolefin matrices where the addition of maleic anhydride-grafted polymers or copolymers [24] as a coupling agent has been identified as one of the most effective methods to achieve a proper dispersion of CNFs [23,25,26,27].

Solvent casting methods can also be applied to nanocellulose dispersion processing. In particular, water-soluble polymers are conveniently used as matrices, as the two components, i.e., polymer and CNFs, can be directly mixed in an aqueous suspension. As a consequence, strong matrix–nanocellulose interactions are ensured due to the polar nature of both the components. For non water-soluble polymers, nanocellulose-based composites preparation via solvent-based techniques is used due to the simplicity of the method at a lab scale. From such a processing, the nanofillers could rearrange to form a percolative network during the slow evaporation of the solvent [6,8,28]. The formation of a stiff filler network also results from the establishment of H-bonding interactions between the nanoparticles [6,8,29]. However, the strong hydrophilic nature of nanocellulose restricts the selection of solvents alternative to water [6]. Several attempts have been undertaken to increase the hydrophobicity of cellulose fillers in order to include non-polar polymers in the set of possible matrices. The two main strategies used can be grouped as the addition of surfactants into the mixture or chemical functionalization of cellulose surface hydroxyl groups [30]. As for chemical functionalization, different processes have been proposed over the years, including functionalization as acetylation [31], esterification [20,21], silanization [32], or grafting of monomers on the cellulose surface [33,34,35]. For these reasons, functionalization of the cellulose can be crucial for the achievement of an adequate matrix–filler interaction. As a consequence, a pre-treatment of the filler is usually required in the preparation of the nanocomposite to guarantee a proper dispersion state.

Despite the necessity of pre-treatments, during the last decade, nanocellulose has been proposed to be used as a filler for a wide variety of matrices, like polyolefins, polyurethanes, polystyrenes, and polymethylmethacrylates [36,37,38]. Polymethylmethacrylate (PMMA) is one of the materials that has been intensively studied for the design of nanocellulose-based composites, thanks to its optical transparency, processability, surface functionalization, and due to the necessity to overcome its main limitations related to an inadequate mechanical strength [33,36,39,40]. Anžlovar et al. grafted methylmethacrylate (MMA) from the CNCs’ surface to improve dispersion in a PMMA matrix, i.e., to reduce CNCs tendency to agglomeration [33]. Jamaluddin et al. reported acylation of CNFs to reinforce PMMA, demonstrating that functionalization with acetyl group enhances interactions with the ester group of a PMMA [36]. Wang et al. demonstrated that a more homogenous dispersion of cellulose nanocrystals can be obtained when the carboxyl groups are grafted on their surface, thanks to the possibility to increase the numbers of H-bond interactions between PMMA and cellulose [41]. In polymer nanocomposites, dispersion and interfacial interactions are the key parameters either to preserve optical transparency or to guarantee a positive effect on the final properties [8,40]. For instance, Dong et al. processed a transparent PMMA nanocomposite reinforced with homogeneously dispersed CNFs (surface functionalized with carboxylic acid). This confirmed that the presence of H-bonds in polymer nanoparticles plays a significant role in the increase in tensile strength as well as the in the achievement of a more ductile behavior compared to the neat polymer [8]. In general, an improvement in mechanical properties of hydrophobic matrices, such as PMMA, is obtained, thanks to the chemical functionalization of the nanofiller surface or the modification of the matrix to guarantee proper interactions and consequently an adequate stress transfer at the interface. For this purpose, cellulose surface is usually chemically modified to decrease its surface polarity, but in a similar manner the introduction of a polar group in non-polar matrices could be beneficial to promote interfacial interactions.

Within the context of the background described above, in this work we propose a copolymer matrix based on poly(methyl methacrylate-*co*-methacrylic acid) (PMMA-*co*-MAA) displaying a dual character, i.e., non-polar and polar. This characteristic is particularly suitable for the dispersion of highly polar fillers, such as nanocellulose, thus contributing to overcome the current limitations in CNF dispersion. Indeed, strong interfacial H-bonding between the matrix and the nanofibers are ensured by the presence of carboxylic acid units. PMMA-*co*-MAA polymer was synthesized via a cobalt-mediated radical copolymerization and the dispersion of CNF was obtained using a simple solvent casting method from a mixture of solvents (tetrahydrofuran/water and tetrahydrofuran/methanol). Such a method simultaneously ensured good solubility of all the components, leading to a proper dispersion of CNFs, as proven via microscopy analyses.

## 2. Materials and Methods

### 2.1. Materials

MMA (99%, inhibited with 30 ppm MeHQ), MAA (99%, inhibited with 100–250 ppm MeHQ), Luperox^®^ DHD-9 (methyl ethyl ketone peroxide MEKP solution, ~32 wt% in phthalate-free plasticizer mixture), tetrahydrofuran (THF, ≥99%, inhibited with 250 ppm BHT) and bis(trifluoromethane) sulfonimide lithium salt (LiNTf_2_, >99%) were purchased from Sigma-Aldrich (Darmstadt, Germany). Lithium chloride (LiCl, 99%, for analysis, anhydrous) was purchased from Acros Organics (Part of Thermo Fisher Scientific, Illkirch-Graffenstaden, France). Methanol (MeOH, anhydrous) and dimethylformamide (DMF, isocratic grade) were purchased from Carlo Erba (Val-de-Reuil, France). Cobalt(II) 2-ethylhexanoate (Accelerator NL-51P, 6 wt% Cobalt in solvent mixture) was purchased from AkzoNobel (Deventer, The Netherlands). CNF water suspension (2 wt%) of standard enzymatic grade was purchased from RI.SE Innventia AB (Stockholm, Sweden). A SEM micrograph in its aggregated state after drying is shown in Figure S1. Deuterated dimethyl sulfoxide (DMSO-*d6*, 99.8%, water < 0.02%) used for nuclear magnetic resonance analyses was purchased from Euriso-Top (Saclay, France). All the products were used as received. Deionized water was obtained from a milli-Q dispenser (Merck Millipore, Burlington, MA, USA).

### 2.2. Synthesis of PMMA-co-MAA

PMMA-*co*-MAA was synthesized in bulk using cobalt-mediated radical copolymerization [42] with a ratio MMA/MAA fixed at 70:30 wt%. Each sample was prepared using 10.50 g (0.10 mol) of MMA and 4.50 g (0.050 mol) of MAA of the as-received monomers with the addition of 0.36 mol of peroxide initiator (MEKP) for 100 mol of total unsaturations (corresponding to 0.12 g and 6 × 10^−4^ mol) and Cobalt(II) 2-ethylhexanoate equivalent to 15 wt% of MEKP (corresponding to 0.02 g and 3 × 10^−4^ mol).

Formulations were stirred for a few minutes to homogenize the composition and then injected with the aid of a syringe in a silicone frame in between two aluminum plates (100 × 100 × 5 mm^3^) defining a 50 × 50 × 5 mm^3^ cavity. Aluminum plates were used as heat sink and dissipator to limit overheating of the mixture during polymerization.

Molds filled with the reactive mixture were then placed in an oven and reaction was thermally activated, thanks to the isothermal steps of 2 h at 55 °C and 70 °C. Complete conversion was ensured via post curing for 2 h at 110 °C and 2 h at 140 °C.

Liquid formulations and corresponding final samples were weighed in order to estimate an average value of the total evaporated mass.

### 2.3. Preparation of PMMA-co-MAA/CNF Nanocomposites

#### 2.3.1. Preparation from THF/Water Suspension

Prior to the CNF dispersion, stability of PMMA-*co*-MAA solution in a THF/water mixture at room temperature was defined. PMMA-*co*-MAA was solubilized in THF and water was gradually added until precipitation occurred.

Pristine PMMA-*co*-MAA films were prepared from THF/water mixtures at different volume ratios (95/5 and 77/23 vol%, both suitable to retain PMMA-*co*-MAA in solution) and in pure THF. The solutions were poured in an aluminum dish and placed under hood for overnight solvents evaporation. Films were further dried under vacuum (30 mbar) for 2 h at 100 °C and compression molded at 230 °C.

For nanocomposites preparation, a given amount of PMMA-*co*-MAA was solubilized in THF overnight and deionized water was added in different amounts (from 0 to 0.1 vol%, calculated to obtain the final THF/water ratio at 77/23 vol%, taking into account of the additional amount of water coming from the addition of the CNF suspension). Next, the CNF aqueous suspension was added into the matrix solution at different contents (from 5 to 25 wt% of nanoparticles in the final composition). The mixture was stirred for 2 h and then poured in an aluminum dish placed under hood to produce films via overnight solvents evaporation. Films were further dried under vacuum (30 mbar) for 2 h at 100 °C, peeled from the aluminum dish and compression molded at 230 °C to remove porosity.

#### 2.3.2. Solvent Exchange

CNF suspension in MeOH was obtained using a CNF aqueous suspension following the procedure described by Roman et al. [43]. Briefly, the CNF aqueous suspension was diluted with the addition of MeOH in a proportion of MeOH/aqueous suspension 3:1 weight. Ultra-Turrax homogenizer T25 basic (Ika Werke, Staufen im Breisgau, Germany, 10 min, room T, 9000 rpm) was used to obtain a dispersion where interactions between CNF and MeOH could be generated. Then, the mixture was centrifugated (Centrifuge 5702, Eppendorf, Hamburg, Germany) for 10 min at 4000 rpm, allowing the precipitation of MeOH-wetted CNFs. The excess of solvents was removed and the residual CNF suspension was subjected to the same procedure for two additional times to ensure complete solvent exchange.

After the third cycle, TGA analysis coupled with IR spectroscopy (TGA 4000 Thermogravimetric analyzer coupled with Spectrum Two IR spectrometer and Red-Shift chiller and transfer line, Perkin-Elmer, Waltham, MA, USA) was carried out on the MeOH-based suspension to verify complete water substitution. The analysis was performed from 50 to 150 °C under nitrogen atmosphere with a heating rate of 5 °C/min.

CNF content in MeOH was calculated from the weight of solid residue after overnight evaporation of the solvent and additional drying in oven for 2 h under vacuum (30 mbar) at 100 °C.

#### 2.3.3. Preparation from THF/MeOH Suspension

In a similar manner to the method described in Section 2.3.1, stability of matrix solution in the mixture of THF/MeOH was defined.

Pristine PMMA-*co*-MAA films were prepared from THF/MeOH at different volume ratios (90/10, 70/30 and 50/50 vol%, all suitable to retain PMMA-*co*-MAA in solution) following the same procedure described in Section 2.3.1.

PMMA-*co*-MAA in a given amount was added into a mixture of THF/MeOH (between 75 and 97 vol% THF) and left to solubilize overnight, where the amount of MeOH varied in relation to the final designed ratio. Next, CNF suspension in MeOH was added as 5, 10 and 15 wt% of filler in the final composition. Films were prepared following the same procedure described in Section 2.3.1.

### 2.4. Characterization Methods

^1^H nuclear magnetic resonance spectroscopy (NMR) (64 scans at 25 or 90 °C and 400 MHz in DMSO-*d6* with Bruker 400 Avance III spectrometer, Billerica, MA, USA) was performed for MMA, MAA and PMMA-*co*-MAA copolymers in order to confirm the ratio between comonomers and calculate the percentage of potential residual monomers. Similar analyses were also performed for the reference film at 25 °C to evaluate the presence of remaining solvents from the manufacturing process. Molar ratio between MMA and MAA was calculated, thanks to the integration of the peaks close to 3.5 ppm and in the region between 0.7 and 1.3 ppm, providing with the following relations.

Area (peak 3.5 ppm) = 3MMA(1)

Area (peaks 0.7–1.3 ppm) = 3MMA + 3MAA(2)

For determining the molar mass of PMMA-*co*-MAA copolymers, either in bulk and film forms, the copolymers were solubilized overnight in DMF + LiNTf_2_ with the addition of 0.75 wt% of LiCl in order to prevent intramolecular H-bonds and facilitate filtration. Then, gel permeation chromatography (GPC) measurements were carried out using a Malvern GPC (Malvern, UK) instrument equipped with a ViscoGEL I-MBHMW-3078 column and a viscosimeter detector. A solution of DMF + LiNTf_2_ was used as an eluent with a flow rate of 0.7 mL/min at 50 °C. Molar masses at the peak distribution (M_n_) and weight-average molecular weight (M_w_) were estimated by applying a calibration method using polystyrene (PS) standards.

Differential scanning calorimetry (DSC) measurements were performed using a TA Q20 instrument (TA Instruments, New Castle, DE, USA), applying a heating ramp of 10 °C/min from −50 to 200 °C under nitrogen atmosphere. For each sample (about 4.5 mg), the glass transition temperature (T_g_) was determined on the second heating ramp trace.

Thermogravimetric analysis (TGA) traces were recorded using TA Instruments Q500 TGA (TA Instruments, New Castle, DE, USA) at 10 °C/min from room temperature to 700 °C under nitrogen or air atmosphere.

Infrared (IR) spectra were collected from 4000 to 400 cm^−1^ via the attenuated total reflectance (ATR) method (16 scans, resolution 4 cm^−1^) using a Nicolet iS10 spectrometer (Thermo Scientific, Waltham, MA, USA) equipped with a diamond crystal. The spectra are reported after being normalized at 1448 cm^−1^.

Transmission UV-vis spectra of the films were recorded via a UV2600 spectrophotometer (Shimadzu, Kyoto, Japan) from 780 to 380 nm using air as blank. From these measurements, absorption was derived, normalized on the film thickness and converted in transmittance %. For a quantitative evaluation of the samples’ transparency, the area under the curves is reported as % of the area corresponding to a fully transparent sample in the analyzed range of wavelengths.

A scanning electron microscope (SEM), EVO 15 SEM Zeiss (Zeiss, Oberkochen, Germany, acceleration voltage 20 kV), and another SEM equipped with a field emission gun (FE-SEM, Zeiss Merlin 4248, Zeiss, Oberkochen, Germany, acceleration voltage 3 kV, probe current 10 pA, working distance 3 mm) were combined to properly investigate the polymethacrylate matrix and nanocomposite films morphologies. The fracture surfaces were obtained after immersion in liquid nitrogen for 3 min to ensure a brittle fracture. The observations were performed on the cross-section of the film after the deposition of a thin gold layer to prevent charging.

Dispersion of CNFs in the matrix was evaluated from transmission electron microscope (TEM, JEM-1400 Flash, JEOL, Peabody, MA, USA) operating at 120 kV as acceleration voltage. For the observation, films were embedded in an epoxy resin and sliced as thin layers (70 nm thick) using Ultramicrotome UC7 (Leica, Nussloch, Germany).

## 3. Results and Discussion

### 3.1. PMMA-co-MAA Copolymer

Cobalt-mediated radical polymerization has been widely described for methacrylic and acrylic monomers, including MMA and MAA [42,44,45]. The MEKP catalyzed via the Cobalt(II) 2-ethylhexanoate (MEKP/Co^2+^) curing system is widely used, especially in unsaturated polyesters resins where styrene or methacrylate are used as reactive diluents [46,47,48]. However, the understanding of the polymerization initiation mechanism remains challenging given the complex MEKP composition and the lack of techniques able to investigate radicals formation with high sensitivity. Therefore, a detailed and complete description of the mechanism has not been discovered yet. The high complexity of the mechanism is also due to the fact that the reaction passes through the formation of numerous intermediate species which will themselves react to form radicals [46]. The present knowledge suggests that the decomposition reaction of MEKP in presence of cobalt salts starts at room temperature and the formation of multiple radical species is observed, mostly methyl, ethyl, and hydroxyl radical [46]. This allows obtaining a copolymer in which acidic units introduces strong polarities along with low polarity MMA units.

**Figure 1:**
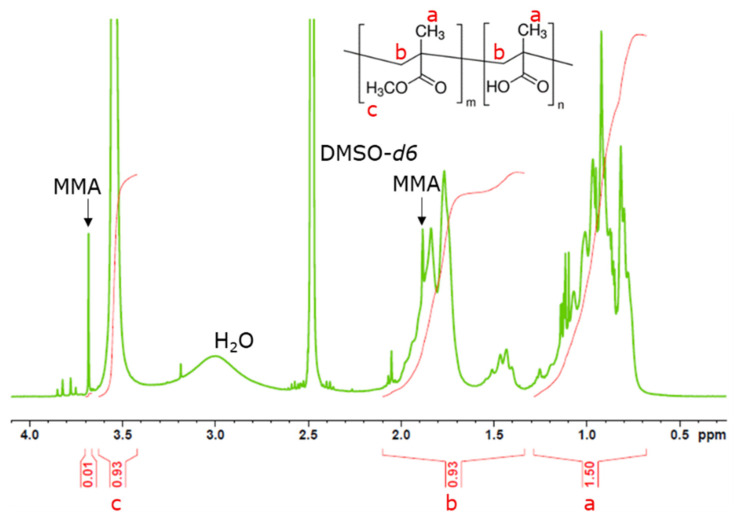
^1^H NMR spectrum at 90 °C for PMMA-*co*-MAA after copolymerization (400 MHz, DMSO-*d6*).

This approach was successfully used in this work for the copolymerization of PMMA-*co*-MAA, resulting in rigid, dense and transparent 5 mm-thick materials (Figure S2). As expected, in the open reactor used, monomer evaporation occurred simultaneously with polymerization. Indeed, the mass loss during copolymerization was calculated to be 11.8 wt% with an initial composition of MMA/MAA 70:30 wt%. Copolymer composition determined via ^1^H NMR (Figure 1; full spectrum is reported in Figure S3a) was found to be 63:37 mol% MMA/MAA [49]. Compared to the expected value (67:33 mol% corresponding to 70:30 wt%), a lower amount of MMA units is obtained, as a consequence of the higher volatility of MMA compared to MAA.

Using ^1^H NMR, it is also possible to identify the resonances corresponding to MMA (shown in Figure S3b at 3.7 and 1.9 ppm). Residual monomer is still present at the final stage. From the integration of the peaks, residual MMA corresponds to 1.5 mol%, while no evidence of residual MAA was found (Figure S3c). Regarding the macromolecular structure, based on the copolymerization constants reported by Krul et al. (*r_MMA_* = 0.84 and *r_MAA_* = 0.62) and their product < 1, it is possible to predict the formation of a random copolymer microstructure [50].

Retention time distribution from GPC shows a relatively broad non-Gaussian peak (Figure S4) at elution time close to the upper limit of the column. The molar masses (M_n_ and M_w_) result in a value much higher than the upper limit identified by the calibration curve of PS, indicating that M_n_ and M_w_ of PMMA-*co*-MAA are higher than 1 × 10^6^ g/mol. The absolute values calculated appear to be poorly reliable owing to the significantly different architecture of PMMA-*co*-MAA compared to PS. However, beside the absolute value, GPC provides evidence for a high molecular weight copolymer with significant polydispersity.

**Figure 2:**
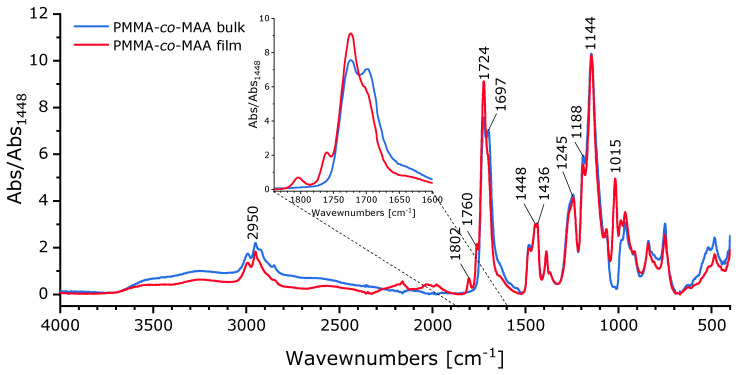
FTIR spectra (ATR mode) of PMMA-*co*-MAA copolymer after copolymerization reaction and film after compression molding at 230 °C for 8 min.

To further investigate the chemical structure of the copolymer, infrared spectroscopy was used. The reported IR spectra are normalized at 1448 cm^−1^ corresponding to the absorption of the bending vibration for CH in α-CH_3_ groups. In the spectrum (Figure 2), it is possible to identify the absorption peaks corresponding to the -C-O-C- vibration at 1144 and at 1188 cm^−1^, -C-C-O- symmetric vibration at 1245 cm^−1^, bending of CH in -O-CH_3_ at 1436 cm^−1^, -C=O symmetric stretching at 1697 and 1724 cm^−1^ and CH stretching close to 2950 cm^−1^ [51]. Focusing on the carbonyl absorption region, the peak at the lower wavenumber (1697 cm^−1^) can be attributed to the acidic units, while the one at 1724 cm^−1^ is related to the ester in MMA. These assignments are in agreement with the results reported by Huang et al. who described the two signals as the superimposition of four signals corresponding to the absorption of the free carbonyls for MAA and for MMA and H-bonded carbonyls, between MMA-MAA and MAA-MAA, respectively. The same authors assigned the peak between 1670 and 1710 cm^−1^ to the carbonyl in MAA forming H-bonds [52]. A shoulder also appears in this range for lower wavenumbers and can be attributed to the presence of water (water bending is close to 1640 cm^−1^) absorbed in the presence of the acidic comonomer [53].

DSC thermogram (Figure S5a) shows a single glass transition temperature (T_g_) at 150 °C, which is higher than the value predicted using the Fox equation:

1/T_g_ = w_1_/T_g1_ + w_2_/T_g2_, (3)

where w_1_ and w_2_ are the weight fractions of the components and T_g1_ and T_g2_ correspond, respectively, to their glass transition temperatures [54]. In fact, the theory predicts a T_g_ equal to 122 °C calculated from the actual copolymer composition and considers the T_g_ of PMMA equal to 104 °C and 170 °C for PMAA, as measured by Huang et al. [52,55]. Moreover, the Kwei equation can also be applied allowing the derivation of a quantitative evaluation of specific interactions present in the system [52,56,57]:

T_g_ = (w_1_T_g1_ + *k*w_2_T_g2_)/(w_1_ + *k*w_2_) + *q*w_1_w_2_, (4)

where *k* and *q* are the fitting parameters. This is the most used equation for blends and copolymers in which strong H-bonds are present. The derived *q* value can be associated with the balance between the breaking of self-associations and the establishment of inter-association interactions. For PMMA-*co*-MMA, *q* result is equal to 121 with *k* fixed to 1, as experimentally reported by Huang et al. for the same copolymer [52,54,56].

The combination of the information issued from DSC thermograms and IR spectroscopy draws attention to the presence of intermolecular H-bond interactions [52]. In fact, the T_g_ exhibited by PMMA-*co*-MAA exceeds the theoretical value by about 30 °C due to the effect of intermolecular H-bonding on macromolecular mobility. As expected, the Fox equation is not able to take into account the additional contribution of such specific and strong interactions. Indeed, the application of the Kwei equation results in a positive value of the *q* parameter suggesting that intermolecular interactions are stronger than the self-association ones as a further confirmation of the presence of strong H-bonds formed between MMA and MAA units. Furthermore, the presence of intermolecular H-bonds is confirmed in IR spectroscopy with the appearance of a peak at 1697 cm^−1^ which can be assigned to the interaction via H-bond involving carboxylic groups [52].

The PMMA-*co*-MAA TGA thermograms (Figure S6) show a weight loss onset (5%weight loss) at 253 °C both under air and nitrogen atmosphere. From the derivative TGA curve in air (Figure S6a), it is possible to identify (i) a first degradation between 200 and 300 °C, (ii) a second one corresponding to the main weight loss centered at 384 °C, and (iii) a last loss starting at 418 °C and ending at 453 °C. The final curve residual weight is <0.5% at temperatures above 500 °C. The derivative TGA curve in nitrogen (Figure S6b) shows (i) a first degradation between 200 and 330 °C, (ii) a main weight loss peak centered at 401 °C, and (iii) a last peak between 490 and 650 °C leading to ~0 final residual weight. From the comparison of the two curves, it is evident that the presence of oxygen has the strongest effect on the last degradation step which shifts to higher temperatures under the nitrogen atmosphere. The details of the main degradation mechanism will be discussed further.

In PMMA-*co*-MAA films analyzed via ^1^H NMR (Figure S3d), it is possible to identify new resonances that do not correspond to the ones of the macromolecule. In fact, the two peaks (1.75–1.78 and 3.59–3.62 ppm) correspond to THF, suggesting that residual THF solvent is still present, while there is no evidence of MeOH (3.17 and 4.10 ppm) [58].

Possible copolymer evolution upon compression molding at 230 °C was evaluated via IR spectroscopy (Figure 2) performed on compression-molded films. Compared to the pristine copolymer, compression molding caused a decrease in the intensity for the peak at 1697 cm^−1^ corresponding to the absorption of MAA groups as well as the appearance of new peaks at 1802, 1760, and 1015 cm^−1^ which correspond to the typical signals for anhydride groups [52].

Furthermore, compression-molded copolymer appears to be insoluble in DMF contrary to the as-synthesized one. This phenomenon confirms the occurrence of a structural evolution and prevents the evaluation of its molar mass via GPC.

DSC analyses performed on PMMA-*co*-MAA film show a single T_g_ at 134 °C (Figure S5b). This value is significantly lower compared to the one at 150 °C found for the pristine copolymer. It is worth mentioning that the T_g_ for compression-molded PMMA-*co*-MAA is closer to the prediction performed using the Fox equation (139 °C). This fact suggests that the condensation of adjacent carboxylic groups may reduce the number of intermolecular H-bonds. Furthermore, the application of the Kwei equation in the case of films pressed at 230 °C leads to a value of *q* equal to 45, which is considerably lower than the value found for the pristine PMMA-*co*-MAA (equal to 121), confirming a partial loss of intermolecular interactions. However, the positive value of the *q* parameter indicates that H-bonds are still present between the chains.

**Figure 3:**
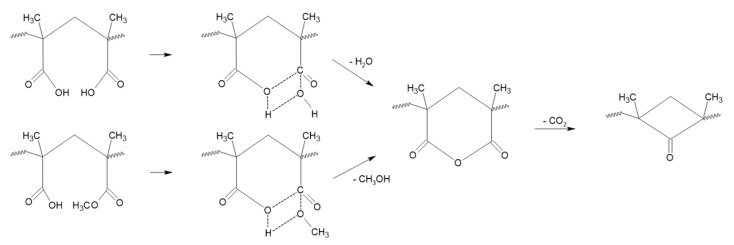
Reaction of anhydride formation via a transition state and decarboxylation of anhydride rings.

From the combination of these results, it is clear that a degradation mechanism occurs when the copolymer is heated at 230 °C for compression molding. Indeed, the changes in the structure can be correlated to the formation of both intra- and inter-chain anhydride groups, as suggested by the change in T_g_, IR spectra, and solubility in DMF. These changes are in agreement with previous conclusions reported by Krul and Jamieson who described the degradation mechanism that occurs in MMA-MAA copolymers [50,59]. PMMA-*co*-MAA copolymers were reported to release volatile products above 220 °C and the chemical species formed can be different depending on the involved paths (Figure 3): while two acidic units react to eliminate a water molecule, when MMA-MAA are adjacent, one MeOH molecule is released. In any case, this condensation reaction leads to the same anhydride structure. The reaction mechanism (Figure 3) is reported only for the intrachain anhydride formation since it corresponds to the favored reaction, however, to a lower extent, it can take place also between interchain units [59].

Above 300 °C, the degradation mechanism refers to the decomposition of the anhydride structure which occurs simultaneously with the beginning of the polymer backbone decomposition via depolymerization. At this stage, different volatile species (CO_2_, CO, and methane) are formed either due to fragmentation of anhydride segments or degradation of the remaining adjacent MMA-MMA units [50,59]. The anhydride rings can partially decarbonize, leading to the formation of a more stable fraction which can be oxidased in air or thermally decomposed under a nitrogen atmosphere as shown via TGA. This mechanism explains the experimental observation described above, with limited temperature differences that can be due to multiple factors, including molar mass and tacticity [60].

Considering the thermal stability of the copolymer, dispersion via melt processing at high temperatures needs to be excluded since it could lead to the elimination of H-bonds and possibly crosslinking. For this reason, a solvent casting route for the dispersion of CNFs is proposed in order to preserve the microstructure of the polymeric matrix and guarantee the formation of copolymer–nanocellulose interactions.

### 3.2. PMMA-co-MAA/CNF Nanocomposite Films

THF is a well-known solvent for acrylics, including the PMMA-*co*-MAA copolymers. However, as THF cannot be used for CNF suspension, solvent mixtures were investigated. THF and water are fully miscible below 27 °C, as reported by Míguez et al. [61]. PMMA-*co*-MAA was found to be soluble in a THF/water mixture with up to 23 vol% of water at room temperature, while precipitation rapidly occurs for higher water contents. Similarly, THF and MeOH are fully miscible at room T and PMMA-*co*-MAA was found to be soluble in the mixture up to a maximum content of MeOH of 90 vol%.

**Figure 4:**
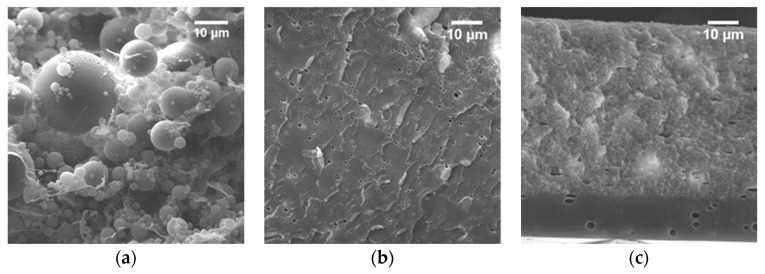
SEM images of PMMA-*co*-MAA + 5 wt% of CNF casted from the THF/H2O 77/23 vol% solution (**a**) and neat PMMA-*co*-MAA casted from the THF/MeOH 70/30 (**b**) and 50/50 vol% (**c**) solutions.

Based on the strong affinity of CNF for water, preparation of nanocomposite films from THF/water was first explored. After the addition of CNF water suspension to the THF/water, copolymer solution and overnight evaporation, opaque nanocomposite films were obtained independently on the CNF contents (5, 15 and 25 wt%). Morphology of the film casted from 77/23 vol% of THF/water polymer solution with 5 wt% of CNF content is reported in Figure 4a. The cross-section exhibits the presence of phase separation with spherical domains up to 25 μm. For a better understating of the phenomenon, the morphology of the neat copolymer matrix was investigated as a function of water fraction in the solvent mixture for three cases: (1) at the solubility limit of the matrix, i.e., in the 77/23 vol% mixture, (2) at the minimum boiling azeotropic point in the atmospheric pressure, 95/5 vol% mixture [62], and (3) in pure THF. From the SEM micrographs, shown in Supporting Information, the copolymer film casted from the higher water content (23 vol%) appears to be stratified in two different regions (Figure S7a), showing a 25 μm thick upper layer characterized by microscopic porosity and a lower layer in the form of homogenous dense phase for the remaining thickness of the sample. The SEM micrograph for lower water content (5 vol% reported in Figure S7b) shows a fracture surface displaying plastic deformation of a homogenous phase. However, after an in-depth investigation, it is possible to identify the appearance of similar spherical domains with a diameter ranging from nanometers up to 3 μm for both the ratios explored. The main difference lies in their amount and distribution. For the higher water content, the phase separated domains are present in large quantities and concentrated in the upper porous layer (Figure S7a), while for the lower content, the analyzed surface shows a homogenous dense phase with few randomly located spherical inclusions (Figure S7c). On the other hand, the copolymer film obtained via solvent casting from the pure THF solution (Figure S7d) shows a homogenous single phase in the whole sample section.

We hypothesize that the phase separation phenomenon occurs due to the large difference in volatility of the two solvents (boiling point is 66 °C for THF and 100 °C for water). As THF has a higher vapor pressure compared to water, the solvent mixture evaporation leads to a progressive increase in water concentration in the solution. With the increase in water concentration, the less hydrophilic polymer chains, i.e., the ones richer in MMA units, are not stable in the solution and will aggregate as particles, whereas the more hydrophilic chains remain soluble and precipitate at a later stage leading to the appearance of a second phase richer in MAA units. Based on the abovementioned mechanism, it is clear that the phase separation observed is driven by a different evaporation rate of the solvents. For this reason, a different solvent mixture was investigated to reduce the difference in volatility of the two solvents. THF/methanol mixture was selected according to the limited boiling point difference (66.0 vs. 64.7 °C, respectively) and based on the possibility to exchange water in the CNF suspension with MeOH, as previously described by Roman et al. [43].

Prior to nanocomposite preparation, the morphology of the neat matrix was investigated for films casted at different THF/MeOH ratios (90, 70, and 50 vol% of THF). The fracture surfaces show a homogeneous morphology characterized by the presence of plastic deformation and pores left from the evaporation of the solvents for the 70 vol% of THF (Figure 4b). Similar results reported in Figure S8 were obtained for a THF content of 90 vol%. On the other hand, the film casted from 50/50 vol% of the THF/MeOH solution (Figure 4c) also presents randomly distributed pores but two different fracture surfaces: a brittle fracture for the lower 1 μm thick layer and a 55 μm thick upper layer with a different fracture behavior, i.e., displaying local plastic deformation.

The solvent exchange process for CNF suspension led to the formation of a visually homogeneous gel in the solvent, with an average CNF content close to 1.8 wt%. The solvent volatilization was completed at 70 °C (Figure S9) and no evidence of water was found via IR spectroscopy, evidencing a successful substitution of water with methanol.

**Figure 5:**
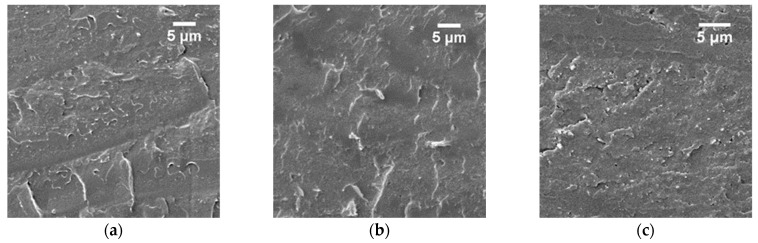
SEM images of PMMA-*co*-MAA + 5 (**a**), 10 (**b**) and 15 wt% (**c**) of CNFs casted from the THF/MeOH 85/15 vol% solutions.

A CNF suspension in methanol was considered for the preparation of nanocomposites in the THF/MeOH solution with solvents ratio fixed at 85/15 vol% and different content of nanofibers (5, 10, and 15 wt%). SEM observations for the PMMA-*co*-MAA nanocomposites based on 5, 10, and 15 wt% casted from the THF/MeOH 85/15 vol% are reported in Figure 5. All the samples clearly appear homogenous and show a fracture surface characterized by the presence of plastic deformation at a submicronic scale. For all the fracture surfaces, the peculiar surface roughness can be correlated to the presence of the nanofibers in the matrix. However, it is possible to identify some smoother areas suggesting that the distribution of CNFs is not fully homogenous, in particular when 10 (Figure 5b) and 15 wt% (Figure 5c) of CNFs are added.

**Figure 6:**
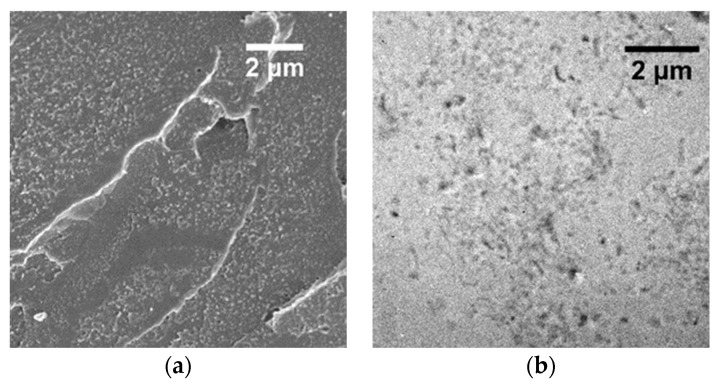

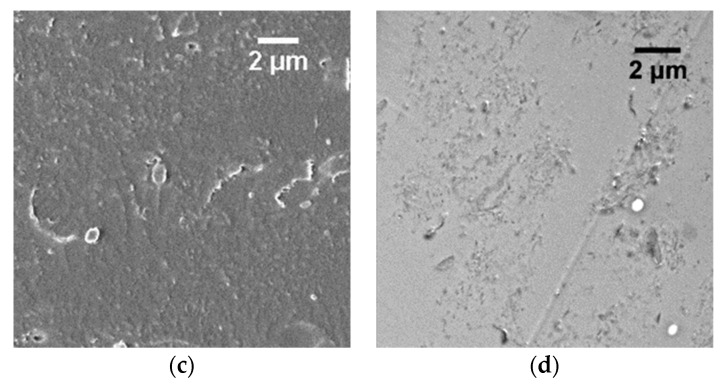
FE-SEM (**a**) and TEM (**b**) micrographs of PMMA-*co*-MAA + CNF 10 wt% nanocomposites processed from the THF/MeOH 85/15 vol% solution. FE-SEM (**c**) and TEM (**d**) micrographs of PMMA-*co*-MAA + CNF 10 wt% processed from the THF/MeOH 66/34 vol% solutions.

The intermediate composition (10 wt% of CNF) was selected to perform an in-depth investigation combining multiple characterization techniques. The dispersion was examined for two different methanol concentrations in the solvent mixture, namely at 15 and 34 vol%, with the maximum value corresponding to the azeotropic mixture at atmospheric pressure [63]. PMMA-*co*-MAA + 10 wt% of CNF casted from the THF/MeOH 85/15 vol% solution was observed at a higher resolution using FE-SEM (Figure 6a), confirming a peculiar surface rugosity that can be associated with the organization of dispersed CNFs within the matrix. To further investigate the dispersion and interconnections of CNFs, TEM analysis was carried out on the same sample and results are reported in Figure 6b. The surface shows different areas with different concentrations of CNFs. However, in the areas at higher concentration, CNFs appear to be dispersed and surrounded by the polymer, indicating good matrix–filler interactions.

Similar analyses have been conducted on films with the same composition casted from THF/MeOH 66/34 vol% solution. From the FE-SEM image shown in Figure 6c, the fracture surface is characterized by a submicronic plastic deformation correlated to the presence of the CNFs within the matrix. From the TEM observations, it is possible to recognize some areas free of nanofibers showing a CNF distribution which is not homogeneous (Figure 6d). However, similar to the film casted from the 85/15 vol% THF/MeOH solution, CNFs appears well dispersed and fully embedded within the matrix.

**Figure 7:**
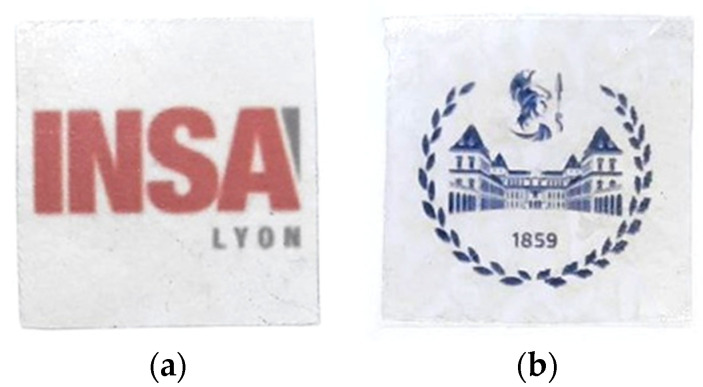
Transparent film of PMMA-*co*-MAA after compression molding at 230 °C (**a**) and with the addition of 10 wt% of CNF after compression molding at 230 °C (**b**).

The solvent casting process leads to the formation of transparent films with the presence of a few white spots related to the presence of pores (Figure S10). After compression molding, all the films are successfully densified and are fully transparent, as reported in Figure 7. Additionally, UV-vis transmittance spectra were acquired to quantify films’ transparency (Figure S11). The spectra show a high value of transmittance in the whole range of wavelengths for all the compositions with a minimum at 98.7%. Quantitative evaluations of the samples’ transparency results in 99.6, 99.5, and 99.1% when 5, 10, and 15 wt% of CNF are added. Their values confirm the optical transparency in the visible wavelength interval for all the samples.

IR-ATR analyses of the transparent films (Figure S12) show new absorption peaks ascribed to the presence of CNF in the matrix: two peaks at 1032 and 1055 cm^−1^ corresponding to C-O-C vibration, a peak at 1105 cm^−1^ corresponding to C-O stretching, and a broad peak from 3060 to 3620 cm^−1^ related to O-H stretching [64]. Furthermore, the peaks corresponding to the formation of the anhydride structure can be recognized in the spectrum at 1802 and 1760 cm^−1^, confirming the occurrence of limited condensation between carboxylic group, as previously observed for the pristine polymer.

DSC thermograms of nanocomposite films for 5, 10, and 15 wt% of CNF (Figure S13) show a T_g_ at 129, 130 and 132 °C, respectively. This suggests that the CNF addition does not influence the glass transition temperature measured via DSC.

## 4. Conclusions

In this study, a methacrylate-based matrix (PMMA-*co*-MAA) was successfully copolymerized in bulk with high degree of conversion and high molar mass. A copolymer was used to prepare the CNF-based nanocomposites. The processing route was based on the solvent casting technique where the dispersion was achieved using a solvent mixture to ensure simultaneous solubilization of the copolymer and dispersion of the CNFs despite their different natures, i.e., non-polar matrix and polar nanofibers, respectively.

Dispersion of CNF nanofibers in the PMMA-*co*-MAA films casted from THF/water displays a phase separation due to different evaporation rates of the two solvents. In order to proceed with the double-solvent mixture approach, MeOH was selected as a substitute for water, thanks to its boiling point closer to the THF and to the possibility to re-dispersed CNFs via a solvent exchanging process. CNF suspension in MeOH was successfully obtained. The solvent mixtureTHF/MeOH, was used to disperse the cellulose nanofibers in PMMA-*co*-MAA. Considering the two solvents selected, the process also has an advantage of high volatile character which facilitates solvents removal via evaporation.

Nanocomposite films were prepared and casted for different THF/MeOH ratios and the dispersion of CNF was evaluated using multiple microscopy techniques. As evidenced via TEM, a good CNF dispersion was obtained from proceeding in the THF/MeOH solution over the range of 85/15 to 66/34 vol% with CNF contents up to 15 wt%. Moreover, FE-SEM microscopy shows that CNFs are embedded in the matrix confirming of good matrix–filler interactions. Therefore, the introduction of a polar comonomer in PMMA chains can ensure a good dispersion of polar fillers, such as CNFs, in a polymethacrylate matrix without nanofibers surface pre-treatment or through the use of a compatibilizer as an interfacial agent. Indeed, the proposed copolymer could be exploited as a compatibilizer in the preparation of PMMA-based cellulose nanocomposites.

## Data Availability

All the data are available upon request.

## Supplementary Materials

The following supporting information can be downloaded at: https://www.mdpi.com/article/10.3390/polym15153226/s1, Figure S1. SEM image of CNF dried from water suspension.; Figure S2. Transparent sample bulk polymerized PMMA-*co*-MAA.; Figure S3. ^1^H NMR spectrum at 90 °C for PMMA-*co*-MAA after copolymerization (a), at 25 °C for MMA (b) and MAA (c), and PMMA-*co*-MAA after compression molding of the film (c) (400 MHz, DMSO-*d6*).; Figure S4. Retention time distribution PMMA-*co*-MAA (30 wt% MAA).; Figure S5. DSC thermograms of pristine PMMA-*co*-MAA showing a T_g_ at 150 °C (a) and after film compression molding at 230 °C showing a T_g_ at 134 °C (b) (heating rate 10 °C/min).; Figure S6. Thermogravimetric traces of PMMA-*co*-MAA in air (a) and in nitrogen (b). Figure S7. SEM images of neat PMMA-*co*-MAA evaporated from THF/water from 77/23 vol% (a), 95/5 vol% (azeotropic composition) (b) with details of spherical inclusions (c) and from pure THF (d).; Figure S8. SEM image of neat PMMA-*co*-MAA casted from THF/MeOH 90/10 vol% solution.; Figure S9. TGA thermogram for CNF water suspension and MeOH-wetted CNF after third washing (5 °C/min).; Figure S10. Residual porosity after solvent casting (THF/MeOH 66/34 vol%) in PMMA-*co*-MAA + 10 wt% CNF film before compression molding.; Figure S11. UV spectra of compression molded films of neat PMMA-*co*-MAA and nanocomposites (5, 10, and 15 wt% of CNF).; Figure S12. ATR spectra of CNF film (a) and PMMA-*co*-MAA + 10 wt% CNF before and after compression molding at 230 °C (b).; Figure S13. DSC thermograms of nanocomposites films of PMMA-*co*-MAA + 5 wt% CNF with T_g_ at 129 °C (a) + 10 wt% CNF with T_g_ at 130 °C (b) and + 15 wt% CNF with T_g_ at 132 °C (c) (heating rate 10 °C/min).
