# Supplementary material for: Dispersion of Cellulose Nanofibers in Methacrylate-Based Nanocomposites
Source: Polymers (Basel). 2023 Jul 28;15(15):3226. doi: 10.3390/polym15153226 (PMC10421470; doi:10.3390/polym15153226)
Supplement: Supplementary file 1 [file polymers-15-03226-s001.zip › polymers-2523249-supplementary.pdf]

# Dispersion of cellulose nanofibers in methacrylate-based nanocomposites - Supplementary information

## Materials

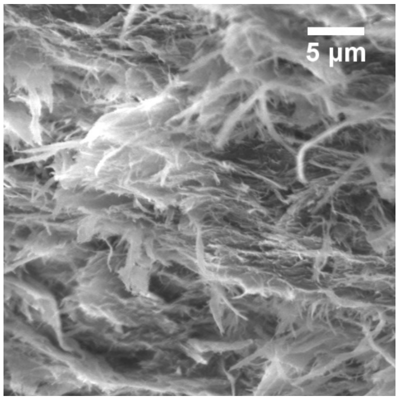


**Figure S1.** SEM image of CNF dried from water suspension.

## Matrix PMMA-*co*-MAA

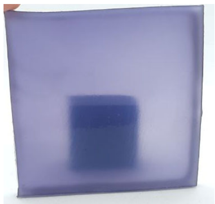

**Figure S2.** Transparent sample bulk polymerized PMMA-*co*-MAA.

**(a)**

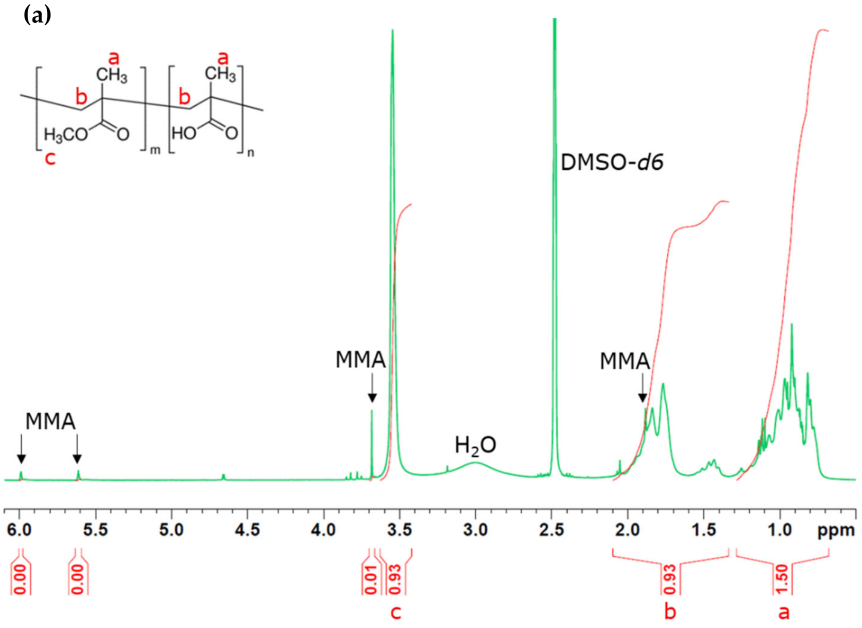

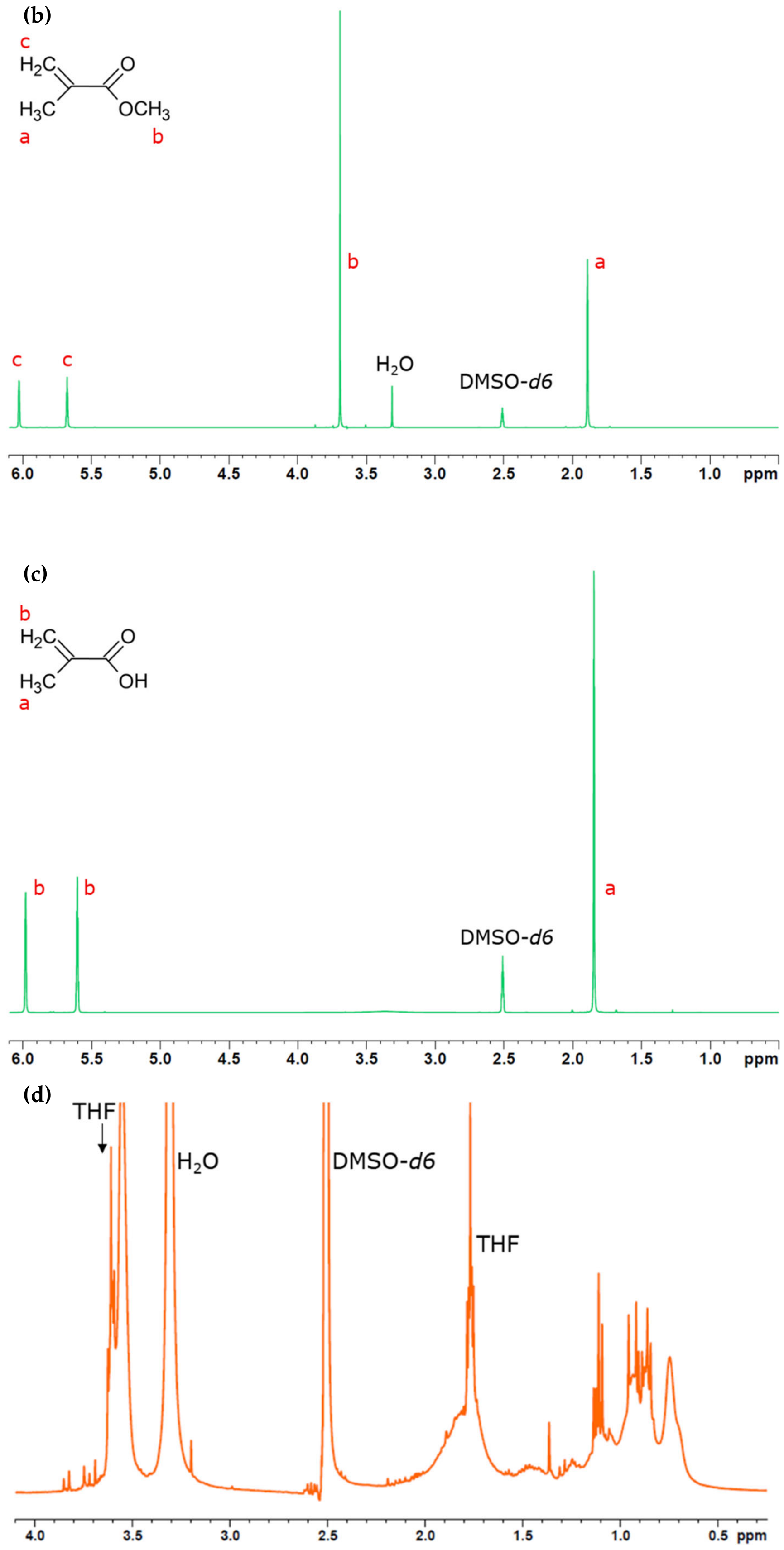


**Figure S3.** $^{1}$H NMR spectrum at 90°C for PMMA-*co*-MAA after copolymerization (a), at 25°C for MMA (b) and MAA (c), and PMMA-*co*-MAA after compression molding of the film (c) (400MHz, DMSO-*d6*).

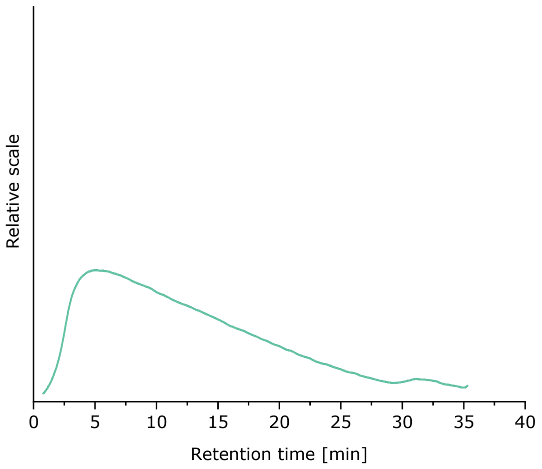


**Figure S4.** Retention time distribution PMMA-*co*-MAA (30wt% MAA).

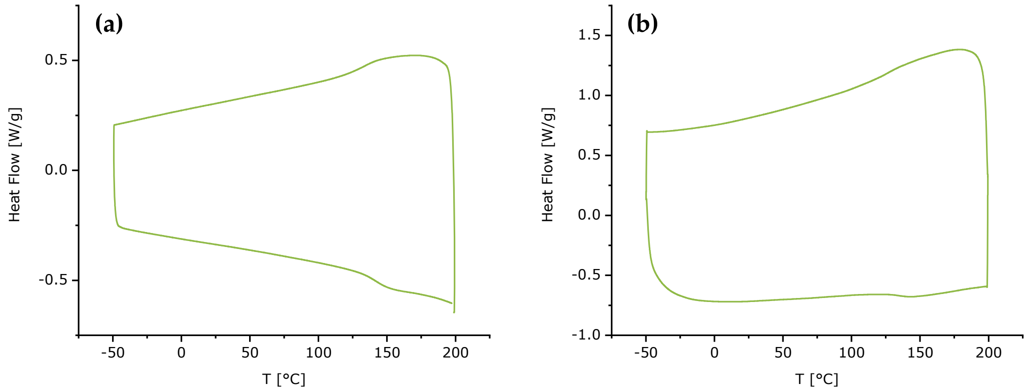


**Figure S5.** DSC thermograms of pristine PMMA-*co*-MAA showing a $T_g$ at 150°C (a) and after film compression moulding at 230°C showing a $T_g$ at 134°C (b) (heating rate 10°C/min).

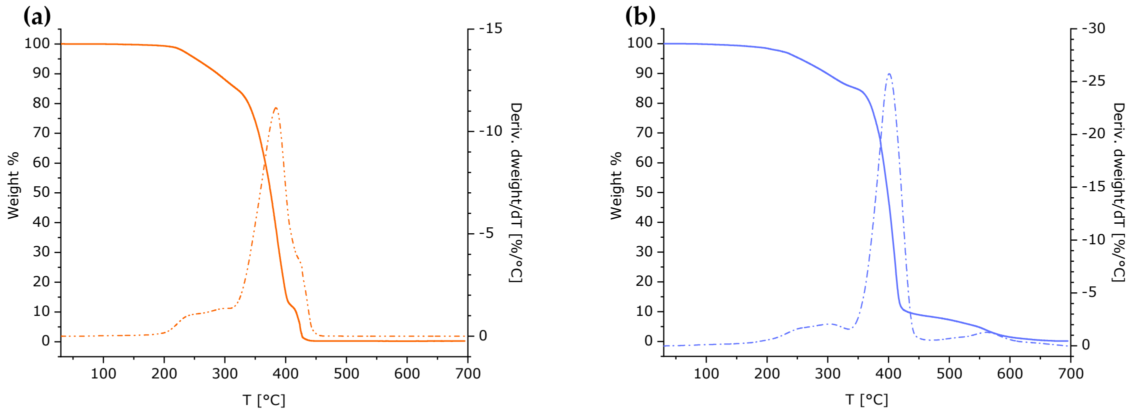


**Figure S6.** Thermogravimetric traces of PMMA-*co*-MAA in air (a) and in nitrogen (b).

## Nanocomposite films from THF/water solution

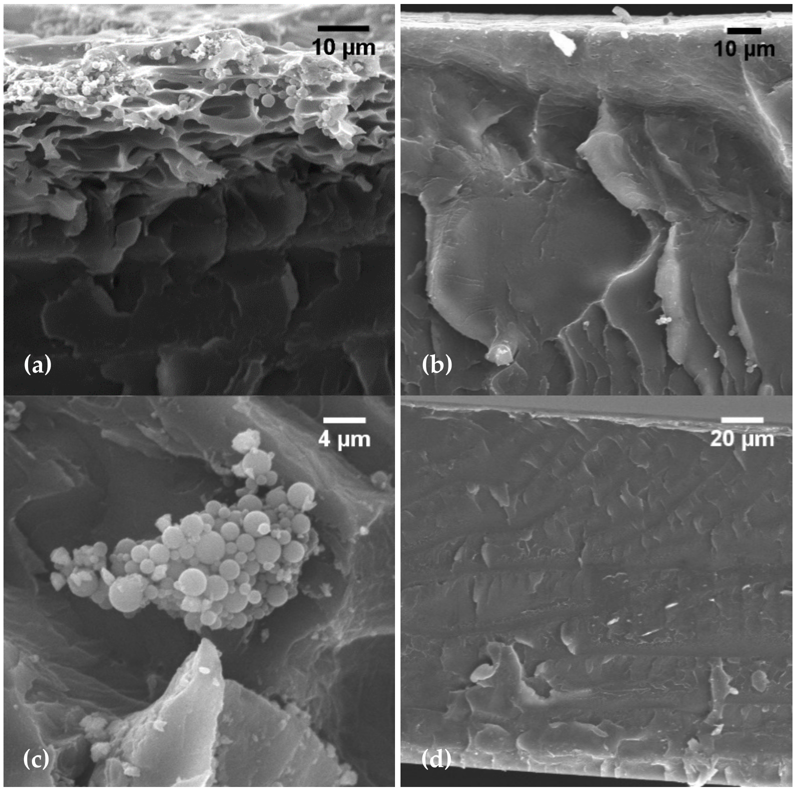


**Figure S7.** SEM images of neat PMMA-*co*-MAA evaporated from THF/water from 77/23vol% (a), 95/5vol% (azeotropic composition) (b) with details of spherical inclusions (c) and from pure THF (d).

## Nanocomposite films from THF/MeOH solution

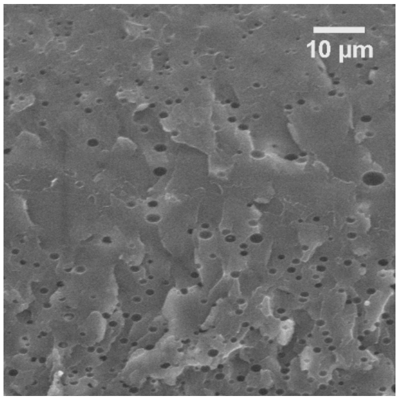


**Figure S8.** SEM image of neat PMMA-*co*-MAA casted from THF/MeOH 90/10vol% solution.

### Solvent exchange

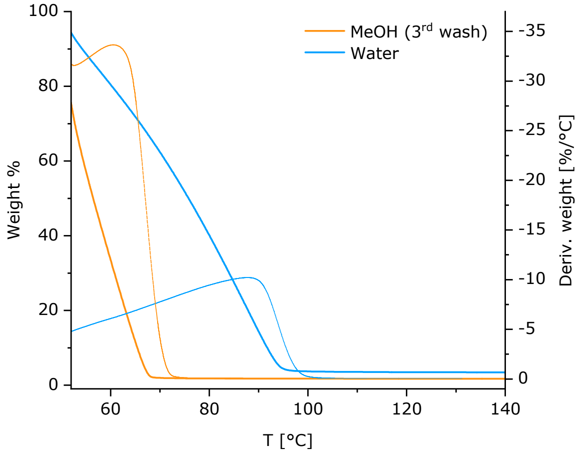


**Figure S9.** TGA thermogram for CNF water suspension and MeOH-wetted CNF after third washing (5°C/min).

### Nanocomposite films from THF/MeOH solution

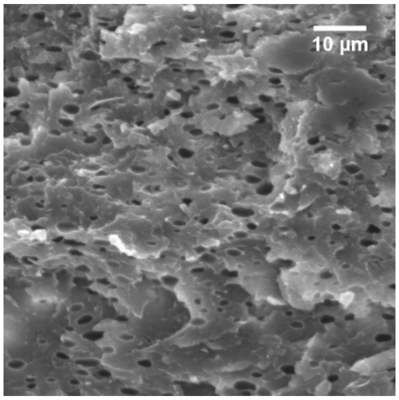


**Figure S10.** Residual porosity after solvent casting (THF/MeOH 66/34vol%) in PMMA-*co*-MAA + 10wt% CNF film before compression moulding.

### Spectroscopy of nanocomposites

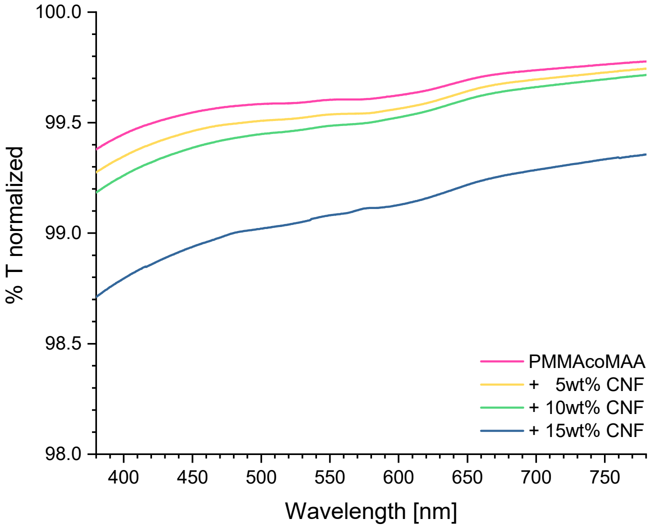


**Figure S11.** UV spectra of compression molded films of neat PMMA-*co*-MAA and nanocomposites (5, 10 and 15wt% of CNF).

CNF film as reference for ATR measurements was prepared by drying CNF water suspension overnight under hood and 2h under vacuum (30 mbar) at 100°C.

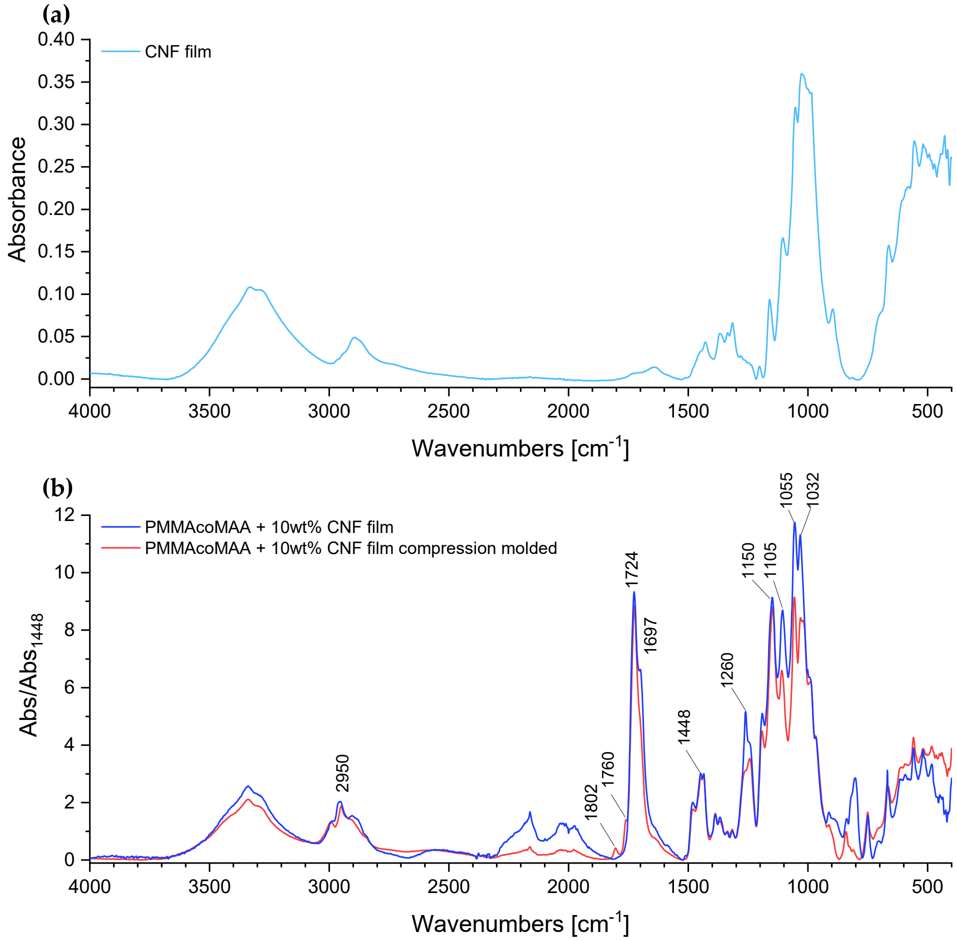


**Figure S12.** ATR spectra of CNF film (a) and PMMA-*co*-MAA + 10wt% CNF before and after compression moulding at 230°C (b).

## Thermal properties of nanocomposites

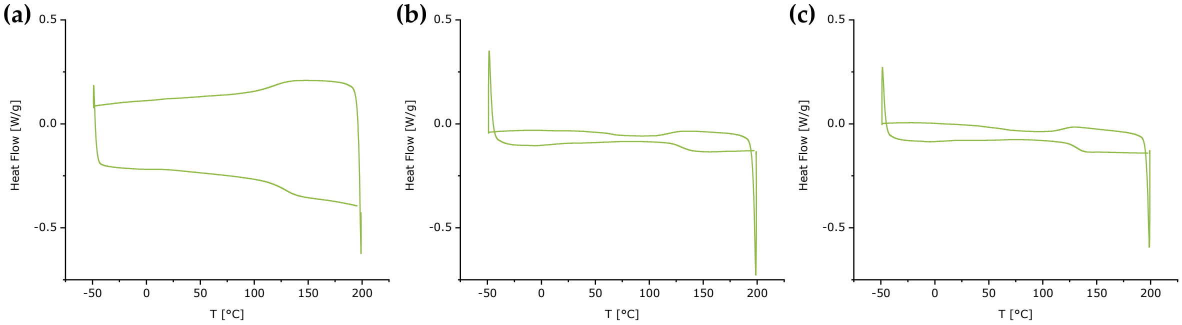


**Figure S13.** DSC thermograms of nanocomposites films of PMMA-*co*-MAA + 5wt% CNF with $T_g$ at 129°C (a) + 10wt% CNF with $T_g$ at 130°C (b) and + 15wt% CNF with $T_g$ at 132°C (c) (heating rate 10°C/min).
